# Changes in Motor Function in a Child with Cerebral Palsy Following Multiple Botulinum Toxin Injections: A Case Report

**DOI:** 10.3390/children12060761

**Published:** 2025-06-12

**Authors:** Nancy Lennon, Chris Church, Jose J. Salazar-Torres, Faithe Kalisperis, Freeman Miller, Jason J. Howard

**Affiliations:** Department of Orthopaedics, Nemours Children’s Health, 1600 Rockland Rd., Wilmington, DE 19803, USA; chris.church@nemours.org (C.C.); jose.salazar@nemours.org (J.J.S.-T.); faithe.kalisperis@nemours.org (F.K.); freeman.miller@gmail.com (F.M.); jason.howard@nemours.org (J.J.H.)

**Keywords:** cerebral palsy, spasticity, botox, motor function, gait

## Abstract

**Objective:** The objective of this study was to examine 7 years of clinical physical therapy measures in a child with spastic diplegic cerebral palsy (CP) who received multiple botulinum toxin type A (BoNT-A) injections. **Methods:** A boy diagnosed with spastic diplegic CP, Gross Motor Function Classification System level II, received four episodes of BoNT-A from ages 4 to 10 years. Serial clinical measures of muscle strength, spasticity, lower extremity passive range of motion, gait kinematics, and gross motor function were collected in the gait analysis lab from age 3 to 10 years. **Results:** After improvements from ages 3 to 7 years, gait and motor function declined from ages 8 to 10 years with no improvement in spasticity or range of motion measurements. Muscle testing and gait kinematics defined a loss of plantarflexion strength. **Conclusions:** A decline in gross motor skills and gait is not typical for a child with spastic diplegia at age 8 years and its association with BoNT-A injections needs to be considered. This case demonstrates the importance of evaluating treatment outcomes for youth with spastic CP utilizing a set of reliable, and clinically useful measures of strength, spasticity, contracture, gait, and motor function. Critical examination of impairment and functional level measures defines goals, guides treatment, and evaluates outcomes. With this approach, pediatric therapists can empower families to make well-informed decisions.

## 1. Introduction

Cerebral palsy (CP) is a non-progressive disorder of movement and posture caused by an early insult to motor centers in the developing brain [1]. Spastic CP, the most common motor type (90%) [2], is associated with hyperreflexia, weakness, and impaired motor control due to disordered corticospinal pathways. Treatments aimed at reducing muscle spasticity are assumed to prevent or delay secondary complications, such as muscle contractures and pathological gait. Children with spastic CP receive a variety of interventions directed at reducing spasticity, promoting optimal postural alignment, and facilitating motor function including physical therapy, orthoses, serial casting, orthopedic surgery, selective dorsal rhizotomy, baclofen therapy, and intramuscular injection of botulinum toxin (the most used serotype being type A [BoNT-A]).

The BoNT-A selectively blocks the release of acetylcholine at the neuromuscular junction resulting in temporary chemo-denervation of the injected muscle [3]. Gradual recovery of neurotransmission occurs, corresponding to approximately 3 months of spasticity reduction [3]. Use of BoNT-A became routine after early reports describing effective short-term treatment for spasticity while being reversible and benign [4,5]. Newer evidence suggests a lack of long-term functional benefit, despite consistent short-term reductions in spasticity [6,7]. A review by Blumetti et al. of evidence for the use of BoNT-A in children with CP found limited evidence that BoNT-A was more effective than placebo or control at improving gait or satisfaction and the results regarding function were contradictory [6]. A review by Ward et al. of evidence supporting tone management in children with CP 0–24 months demonstrated a lack of high-level evidence support [7].

Additionally, a review of BoNT-A interventions highlights the induction of sustained muscle atrophy (i.e., sarcopenia) and fibrofatty tissue replacement post injection in animal models and human imaging studies [3]. The review by Multani et al. (2019) provides a mechanism for clinicians to increase awareness of potential adverse events related to muscle injection treatments [8]. Several recent studies in animal models and humans using advanced imaging and histologic evaluation confirm that intramuscular injection of BoNT-A in standard therapeutic doses effects muscle in ways that go beyond temporary denervation [8]. Acute muscle atrophy is noted by reductions in muscle cross-sectional area [8]. The volume of contractile elements is reduced, and collagen-based tissue is increased and accompanied by disordered muscle tissue structure [8]. The inflammatory molecules, proteinases, adipokines, and mesenchymal stem cells increase after BoNT-A injection, which limits muscle recovery and promotes fatty tissue infiltration [8]. Acute inflammatory response is also seen in upregulation of interleukin-6 with increased collagen synthesis from BoNT-A-induced muscle damage [8]. These changes at the molecular level are associated with well documented reductions in muscle torque and loss of contractile function [8]. Future research needs to examine long-term outcomes of BoNT-A in larger cohorts utilizing multiple outcomes, including muscle imaging, muscle histology, muscle performance, and motor function to evaluate the acute and long-term effects of BoNT-A and similar agents. Despite an established safety profile from a systemic point of view, these findings raise concerns that BoNT-A is not completely benign and may result in adverse effects localized to the muscle including exacerbation of weakness [8].

The prospect of utilizing a clinical intervention that induces muscle weakness is concerning, as muscle weakness is a primary determinant of gross motor function in CP [9]. The lower extremity muscle strength of children with CP is 36% to 82% of typically developing peers [10]. The potential for BoNT-A injections to exacerbate lower extremity muscle weakness and reduce gait or motor function needs closer attention by clinicians.

## 2. Case Description

The patient was referred to a CP specialty clinic housed in a tertiary pediatric hospital at age 2 years. Chart review describes a history of birth prematurity and neonatal complications. He was born at 24 weeks gestation via cesarean section with a birth weight of 595 g. He stayed in the newborn intensive care unit for 120 days and was intubated for 2 months postnatally. Complications included respiratory distress syndrome, surgically corrected patent ductus arteriosus, grade 3 intraventricular hemorrhage, and retinopathy of prematurity.

At age 2 years, he was sitting independently, non-reciprocally creeping around the house, and pulling to stand. With hands supported, he could take steps on his tiptoes with his knees flexed. At that time, he was receiving physical therapy (PT), occupational therapy, and speech services through local early intervention. He continued to receive specialty medical care through the comprehensive multidisciplinary CP clinic with follow-up every 6 to 12 months. During regular visits to the CP clinic, therapy programming was reviewed, adaptive devices were checked, and gait laboratory data were collected. His therapy care was through local early intervention, school programming, and, after BoNT-A injections, outpatient PT at a therapy center near the family’s home.

At age 4 years, he walked with a rear walker and could creep on his hands and knees. Neuro-orthopedic concerns at that time included hamstring spasticity, fixed hamstring contracture, and Rodda gait classification of “apparent equinus” [11], a “heel-off” gait driven by excessive knee and hip flexion with the ankle in neutral dorsiflexion. At age 4.5 years, with goals of more upright posture, plantigrade foot position, and stability during stance, he received BoNT-A injections of 50 units each to both hamstrings. During the 8 weeks after injection he received a bout of PT at a local clinic to work on hamstring flexibility and gross motor mobility. Soon after, at age 5 years, he began taking independent steps. Parents reported continued improvements in gross motor skills through age 7 years, including independent transitions to and from the floor, stepping up and down curbs, and fast walking. This progression of skill development is consistent with the development of gross motor function in youth with CP as described by Hanna and colleagues [12,13].

At age 8 years, the child’s family sought a second opinion from a separate pediatric specialty hospital, motivated by a desire to avoid orthopedic surgery. From age 8 to 10 years, he received care at both institutions. A physiatrist at the second institution recommended BoNT-A injections. At age 8 years, the patient received injections to bilateral hip adductors, hamstrings, and gastrocnemius in a total dose of 300 units. The same BoNT-A protocol was repeated at age 9 and 10 years, consistent with common clinical practice at that time [5]. Following each episode of BoNT-A, the patient engaged in a bout of outpatient PT for flexibility and gait training activities. Details of PT were not available in the hospital chart review.

The two institutions providing care to this patient had different approaches to the management of muscle contractures and spasticity in youth with CP. The first center favored fewer interventions to reduce spasticity and instead promoted therapy, orthoses, and gait monitoring, with consideration for orthopedic surgery when gross motor skill development plateaued. The second institution had a common practice of repeat BoNT-A injections to treat dynamic (i.e., spastic) muscle contractures throughout childhood.

### 2.1. Clinical Measures

Physical exam and kinematic collection were repeated over several years by the same physical therapist during multiple visits to the gait analysis lab at the first center. The Commission for Motion Laboratory Accreditation granted accreditation status to this lab, which demonstrates achievement of consistency in measurement protocols. Reliability of serial measurements is essential in the evaluation of change following intervention [14]. Medical records from the second center did not report spasticity, passive range of motion (PROM), strength, gross motor function, or gait parameters.

Goniometric measures in youth with CP demonstrate fair reliability, with test-retest standard error of measurement of 4° to 5° for ankle dorsiflexion and 3° to 9° for popliteal angle [15]. The Modified Ashworth Scale (MAS), a clinical tool rating resistance to quick stretch on a 0 to 4 scale, demonstrates acceptable reliability with intra-class correlation coefficient of 0.56 (range, 0.33–0.78) for single raters for the same participant [16]. Manual muscle testing (MMT) is used clinically and when performed by the same physical therapist, MMT grades have good reliability (range, 0.80–0.99) [17]. The Gross Motor Function Measure (GMFM) has strong psychometric properties when used for youth with CP, including validity with motor classification (rho 0.72–0.77) and responsiveness to change, with standard error of mean and smallest real difference of 1.60 and 3.14 [15,18]. Instrumented gait kinematics are a valid and reliable tool, widely used to document gait deviations and evaluate change, with standard error of mean in the range of 3° for lower extremity models [19].

### 2.2. Outcomes

Early in this patient’s neuro-orthopedic management, prior to BoNT-A injections, observational assessment of gait revealed excessive knee flexion during stance associated with normal ankle dorsiflexion. At age 3.5 years, there were high MAS scores of hamstring spasticity, associated with fixed contracture, as well as very mild gastrocnemius spasticity and normal ankle dorsiflexion (Table 1). These findings corroborate the Rodda gait classification of apparent equinus driven by hamstring spasticity or contracture [11]. At that stage, BoNT-A injections to the hamstrings were based on clinical findings and parental goals of independent gait. The outcome measures at the impairment and functional level were improved at age 5.5 years, with better hamstring flexibility, reduced hamstring spasticity, and gains in the GMFM, including achievement of independent walking. These GMFM gains, however, should be expected by natural history, even without treatment, during this phase of motor development.

**Figure 1:**
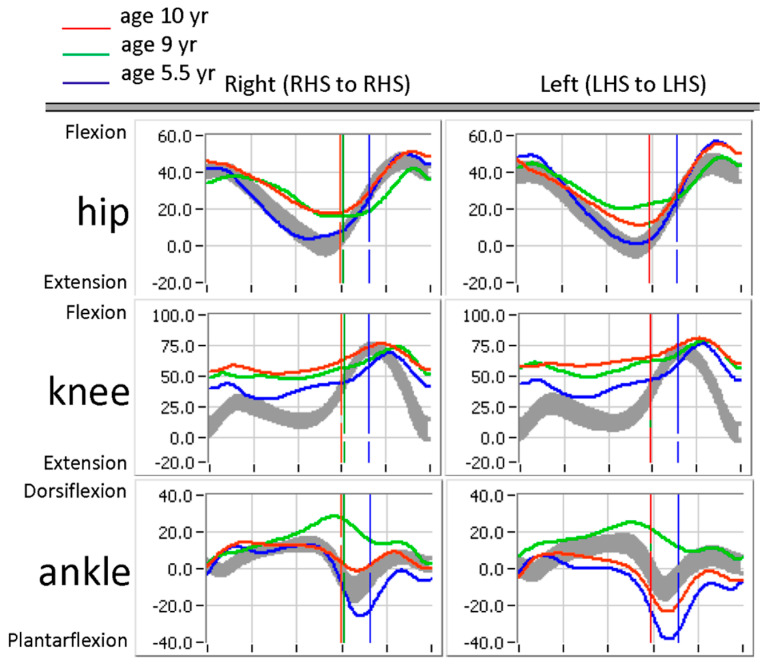
Sagittal plane hip, knee, and ankle kinematics. LHS, left heel strike; RHS, right heel strike (gray band-9 yr old normal).

The patient received two larger doses of BoNT-A to multiple muscle groups at ages 8 and 9 years. The clinical examination measures prior to these injections do not support treatment of the hip adductors and gastrocnemius, where spasticity measures were relatively mild (Table 1) [5]. By age 9 years, stiffness in the hip adductors increased despite BoNT-A injections. Hamstring spasticity and PROM did not change, and gastrocnemius contracture (relative to soleus) worsened. The MMT measures had no meaningful change; however, gait kinematics showed a loss of controlled “second rocker” (i.e., mid-stance dorsiflexion) at the ankle, possibly related to functional calf weakness seen on physical exam. The excess dorsiflexion during this phase of the gait cycle likely contributed to excess knee flexion and hip flexion. This pattern progressed quite dramatically from age 5.5 years (Figure 1).

After the fourth round of BoNT-A injections at age 10 years, the MAS ratings peaked, and the degree of fixed muscle contractures was clinically concerning and unimproved. At this stage, there was a clear loss of strength in the ankle plantar flexors according to MMT. Gait kinematics showed persistent crouch with excess hip and knee flexion, although ankle dorsiflexion was less severe. There was a loss in GMFM Dimension D score, from 76% to 69%, and most concerning, the family reported that the patient started crawling rather than walking around the house.

## 3. Discussion

The purpose of this case report is to describe changes in strength, PROM, gross motor function, and gait seen in a boy with spastic diplegic CP treated with BoNT-A injections over 7 years of childhood. This report provides an opportunity to examine long-term changes of the body systems and body function levels of the International Classification of Functioning, Disability, and Health and consider whether this treatment supported the patient and family’s activity and participation goals.

While the relatively long intervals could not capture the short-term improvements in spasticity or PROM that would be expected immediately after BoNT-A injections, there were no sustained improvements in spasticity or reduction in fixed contracture. Rather than decreasing, muscle stiffness peaked at age 10 years after the four rounds of BoNT-A. Similarly, PROM gains were only seen after the first hamstring injections, which may have been associated with improvements in independent gait. In subsequent years though, hip adductor, hamstring, and gastrocnemius contractures all worsened. The ankle PROM measures indicated that while gastrocnemius muscle length was short, soleus length was excessive. This pattern of contracture, especially in combination with calf weakness, can induce crouch gait in youth with spastic CP [20]. The trajectory of spasticity measures for this patient go against the natural trend in spasticity described by Häggland and Wagner, with an early peak between ages 3 and 5 years followed by gradual decline until adolescence [21]. Rather than reducing spasticity, the multiple doses of BoNT-A are suspected of contributing to increased muscle stiffness in this patient [3].

The “temporary” weakness following BoNT-A described earlier [22] was not the case for this patient. The change in ankle plantarflexion MMT scores and excessive ankle dorsiflexion in gait kinematics suggest a loss of muscle strength in the gastrocsoleus over time. Calf weakness is associated with progressive crouch gait and, for this patient, may explain the dramatic reduction in walking speed and regression to crawling at age 9 years. While calf weakness appeared to progress in this patient, quadriceps strength was maintained or improved. This could be due to a proximal compensation to maintain upright posture with use of the quadriceps to counteract the tendency to crouch. This pattern is associated with high energy cost and fatigability [23].

Finally, GMFM change and slower gait speed represent objective losses in International Classification of Functioning, Disability, and Health measures of activity capacity while the regression to crawling was a dramatic decline of participation.

Although the long-term objective measurements by the same clinician are a strength of this case study, the lack of short-term measurements, taken immediately after BoNT-A, limit the assessment of temporary treatment effects. More comprehensive strength testing using dynamometry versus MMT could have better captured a progression of muscle weakness. Given emerging data reporting increased muscle atrophy and fibrofatty muscle replacement in animal models and human imaging studies, future research needs to examine long-term outcomes of BoNT-A in larger cohorts and should utilize advanced imaging and histology to evaluate the effects of BoNT-A on muscle structure.

The situation in which this family chose to seek care at two separate institutions presented a dilemma to providers. The two centers had different philosophies for neuro-orthopedic care. Pediatric therapists could play an important role in helping families negotiate such situations. Physical therapists who are actively involved in a care plan should collaborate with families in researching treatment recommendations and discussing functional goals and measurement methods.

## 4. Conclusions

This case demonstrates the importance of evaluating treatment outcomes for youth with spastic CP utilizing a set of reliable and clinically useful measures of strength, spasticity, contracture, gait, and motor function. These serial measurements captured declines over the course of repeated treatment with BoNT-A injections in this patient.

## Figures and Tables

**Table 1:** Longitudinal Data on Spasticity, Contractures, Strength, Gross Motor Function, and Gait.

BoNT-A Timing & Muscle Groups	Pre-BoNT-A		15 Months Post (HS Only)		6 Months Pre-BoNT-A		7- & 1-Month Post (BoNT-A: ADD-HS-GS ×2)		3 Months Post (BoNT-A: ADD-HS-GS)	
Patient age	3.5 years old		5.5 years old		8 years old		9 years old		10 years old	
Height (cm) Weight (kg)	97 12.8		102 17.4		120 21.1		123 24.7		130 26.7	
Side	R	L	R	L	R	L	R	L	R	L
Spasticity (MAS)										
Hip adductors	2	2	1+	2	1+	1+	3	3	3	3
Hamstrings	3	3	1	1	2	2	2	3	2	3
Gastrocnemius	1	1	1+	1+	1+	2	1+	1+	3	3
PROM										
Hip extension	−5°	−5°	10°	10°	−5	−5	−5°	−10°	−5°	−5°
Hip abduction	12°	15°	15°	20°	20	18	15°	13°	13°	16°
Knee extension	−10°	−10°	−11°	−7°	−10	−15	−11°	−15°	−13°	−21°
Popliteal angle	100°	90°	68°	70°	75	80	75°	75°	78°	80°
DF (knee flexed)	35°	25°	15°	15°	20	0	20°	15°	22°	15°
DF (knee extension)	10°	10°	10°	0°	10	−8	−3°	−7°	0°	−5°
Strength (MMT)										
Hip extensors	m	m	3+	3+	3+	3+	4-	4-	4	4
Knee extensors	m	m	3+	3+	3+	3+	4	4	4+	4+
Knee flexors	m	m	3+	3+	3+	3+	4-	4-	4	4
Ankle plantar flexors	m	m	3+	3+	3+	3+	3-	3-	2	2
Kinematics (maximum values during stance)										
Hip extension	m	m	−3°	0°	m	m	−17°	−20°	−18°	−13°
Knee extension	m	m	−30°	−32°	m	m	−48°	−50°	−50°	−60°
Ankle dorsiflexion	m	m	10°	5°	m	m	28°	23°	14°	7°
Gross Motor Function										
GMFM-D	10/39		23/39		30/39		30/39		27/39	
Walking speed	m		75 cm/s		m		32 cm/s		76 cm/s	

m indicates missing data. ADD, adductors; BoNT-A, botulinum toxin type A; DF, dorsiflexion; HS, hamstrings; GS, gastrocnemius; GMFM-D, Gross Motor Function Measure Dimension D, MAS, Modified Ashworth Scale; MMT, manual muscle testing; PROM, passive range of motion; R, right; L, left.

## Data Availability

All relevant data for this case study is contained within this paper.

## Abbreviations

The following abbreviations are used in this manuscript:

BoNT	Botulinum toxin type A
CP	Cerebral palsy
GMFM	Gross Motor Function Measure
MAS	Modified Ashworth Scale
MMT	Manual muscle testing
PROM	Passive range of motion
PT	Physical therapy
